# Clinical profile of bloodstream infections in COVID-19 patients: a retrospective cohort study

**DOI:** 10.1186/s12879-021-06647-x

**Published:** 2021-09-08

**Authors:** Naveenraj Palanisamy, Nakka Vihari, Durga Shankar Meena, Deepak Kumar, Naresh Midha, Vibhor Tak, Ankur Sharma, Gopal Krishana Bohra, Nikhil Kothari, Naveen Dutt, Pradeep Kumar Bhatia, Mahendra Kumar Garg, Sanjeev Misra

**Affiliations:** 1 Department of Medicine, All India Institute of Medical Sciences, Jodhpur, Rajasthan 342005 India; 2 Department of Microbiology, All India Institute of Medical Sciences, Jodhpur, 342005 India; 3 Department of Anaesthesia and Critical Care, All India Institute of Medical Sciences, Jodhpur, 342005 India; 4 Department of Pulmonary Medicine, All India Institute of Medical Sciences, Jodhpur, 342005 India; 5 All India Institute of Medical Sciences, Jodhpur, 342005 India

**Keywords:** COVID-19, Pneumonia, ICU, Antimicrobial resistance, BSIs, *Acinetobacter baumannii*

## Abstract

**Background:**

Bloodstream infections (BSIs) are an emerging cause of significant morbidity and mortality in severe Coronavirus disease 2019 (COVID-19). We aimed to assess the prevalence, clinical profile and outcome of BSIs in critically ill COVID-19 patients.

**Methods:**

This was a single-centre retrospective study conducted at a tertiary care hospital in Western India. All patients (age > 18 years) with reverse-transcription polymerase chain reaction (RT-PCR) confirmed COVID-19 admitted in the intensive care unit (ICU) were included. Hospital electronic records were searched for demographic data, time of bloodstream infection since admission, clinical profile, antimicrobial resistance pattern and clinical outcome of all patients who developed BSIs.

**Results:**

Out of 750 patients admitted in COVID ICU, 8.5% developed secondary BSIs. All severe COVID-19 patients who developed BSIs succumbed to illness. A significant proportion of BSIs were Gram-negative pathogens (53/64, 82.8%). *Acinetobacter baumannii* was the commonest isolate, followed by *Klebsiella pneumoniae* (32.8% and 21.9%, respectively). Multidrug-resistance organisms (MDRO) were found in 57.8% of the cases. The majority of MDRO belonged to *K. pneumoniae* and *Enterococcus* groups. The proportion of Gram-negative bacteria resistant to carbapenems was 47.2% (25/53). On multivariate analysis, raised total leukocyte counts, mechanical ventilation and presence of comorbidities were significantly associated with the incidence of BSIs.

**Conclusion:**

We found a significant prevalence of *Acinetobacter baumannii* in COVID-19 associated BSIs. The presence of comorbidities raised leukocyte counts and mechanical ventilation should alarm clinicians for possible BSIs. The timely initiation of empirical antibiotics and rapid de-escalation is vital to improve the outcome. At the same time, strict compliance of infection control practices should be accomplished to reduce the occurrence of MDRO.

## Background

The COVID-19 pandemic is an ongoing public health crisis causing the death of more than three million people worldwide at the end of May 2021 [1]. Critical COVID-19 is reported in around 5% of the cases, which requires intensive care admission [2]. The case-fatality rate is highly variable (1.39–14%) depending on the demography of infection, clinical standard, and epidemic wave dynamics [3]. During the course of hospitalization, it is difficult to predict the secondary bacterial infections, which warrant the use of empirical antimicrobials in patients with severe COVID-19. Recent reports show conflicting results regarding the prevalence of secondary bacterial infections (ranges from 14.3 to 67.7%), particularly bloodstream infections [4–8]. In addition, previous reports have shown prolonged hospital stay, morbidity, and mortality (odds ratio = 3.31, 95% CI 1.82–5.99) in the presence of bacterial superinfections [9, 10]. There is a scarcity of data from India regarding the BSIs and their impact on mortality in COVID-19. Knowing the local epidemiology and the impact of BSIs is paramount in order to apply prompt management and guide an empirical antimicrobial therapy when clinically appropriate. We aim to assess the prevalence, clinical profile, risk factors, frequency and distribution of microorganisms, antimicrobial susceptibility, and clinical outcome in severe COVID-19 with BSIs.

## Methods

This retrospective observational study was carried out at a tertiary care centre in western Rajasthan, India, in a dedicated COVID-ICU referral centre. From July 2020 to December 2021, all patients with confirmed COVID-19 (RT-PCR positive on nasopharyngeal or oropharyngeal swab) who were admitted to COVID-19 ICU were included. Only patients who developed BSIs 48 h after the hospital admission were included. Patients who already had BSIs at the time of current admission were excluded. The study was approved by the institutional ethical committee (reference no -AIIMS/IEC/2020/3174). Bloodstream infections were defined by the presence of viable bacterial or fungal microorganisms in the bloodstream (demonstrated by the positivity of one or more blood cultures) that have elicited an inflammatory response characterized by the alteration of clinical, laboratory, and hemodynamic parameters [11]. For skin contaminants (like coagulase-negative Staphylococcus), at least two consecutive blood cultures for the same pathogen were considered as BSIs. Isolation of the same microorganisms from the bloodstream within 14 days was not considered a novel event and excluded from the analysis. Blood cultures were usually sent in patients with persisting fever (> 38.3 C), raised leukocyte counts, clinical signs of new septic foci, and clinical deterioration after initial improvement. Regarding central venous access, we have tried to minimize it in a view of possible central line-associated bloodstream infections (CLABSI). The central line was inserted only in cases with mechanical ventilation and difficult peripheral access.

All electronic health records were searched, and the following clinical data were extracted: Demographic parameters (age, gender), risk factors for hospital-acquired BSIs like indwelling catheters, mechanical ventilation, use of immunosuppressants (corticosteroids or Tocilizumab for primary disease), comorbidities (end-stage renal disease, chronic liver disease, or malignancy), use of antibiotics, the timing of blood culture drawn, duration of ICU stay, and outcome of patients. Haematological and biochemical parameters were collected, including haemoglobin, total leukocyte count, platelet count, c-reactive protein (CRP), procalcitonin, and lactate dehydrogenase (LDH). Quick sequential organ failure assessment (q-SOFA) score at baseline (at the time of ICU admission) was collected for each patient. Septic shock was defined with persisting hypotension requiring vasopressors to maintain MAP ≥ 65 mm Hg and having a serum lactate level > 2 mmol/L (18 mg/dL) despite adequate volume resuscitation [12].

### Identification of BSIs and antimicrobial susceptibility pattern

Eight to ten ml of blood samples were collected aseptically and were inoculated into the BACTEC (BD, Biomerieux, France) bottles. The bottles were then loaded onto the automated system and incubated for 5 days minimum. Upon flagging positive, the bottles were taken out of the instrument, and the time to detection (TTD) was noted. All those bottles which did not flag positive within five days were considered sterile. The positively flagged bottles were subjected to Gram’s stain and culture onto blood agar and MacConkey agar. After 16–18 h of incubation at 37 °C, the cultures were read for the growth of colonies and the Gram’s stain and catalase tests were performed for further work-up. Identification of microorganisms in the majority of the cases was made by an automated VITEK-2 system. Antibiotic susceptibility testing was performed by Kirby Bauer’s disk diffusion method. The zone of inhibition for different antibiotics is compared to the criteria set by the Clinical and Laboratory Standards Institute (CLSI) [13]. Multidrug-resistant organism (MDRO) was defined as acquired non-susceptibility to at least one agent in three or more antimicrobial categories [14]. CR-BSIs cases were defined as a carbapenem-nonsusceptible and extended-spectrum cephalosporin-resistant (e.g. ceftriaxone, ceftazidime, ceftizoxime, and cefotaxime) isolates recovered from bloodstream [15].

### Statistical analysis

Data analysis was performed using SPSS software, version 20.0 (IBM Corp, Armonk, NY). Descriptive data were summarized and tabulated with continuous variables in the form of mean ± standard deviation, Median (inter quartile range) and categorical data in the form of percentages or frequencies. Linear regression model was used to highlight the association between day of culture positivity (duration from the day of hospitalization to blood culture positivity) and mortality. Coefficient of determination (R square) was calculated to show the percentage of variation in mortality attributed to day of culture positivity. Univariate analysis was performed to identify the association between various factors and incidence of BSIs. We then conducted a multivariate analysis of variables that were found to be significantly associated with Covid-19 with a similar threshold of significance (p-value < 0.05).

## Results

During the study period of 6 months (July 2020–December 2021), a total of 750 patients with RT-PCR confirmed COVID-19 were admitted to ICU. Overall, 8.5% of patients (64/750) developed BSIs. Demographic and clinical characteristics of all patients (BSIs and non-BSIs) are summarized in Table 1. The median age of all patients with BSIs was 65 years (IQR 54–70). The demographic features (age, gender) did not differ significantly between BSIs and non-BSIs patients (Table 1). The median qSOFA score was 2. Significant prevalence of comorbidities was found in BSIs patients compared to non-BSIs (70% vs 39.7%). Among the comorbidities, hypertension, diabetes, ischemic heart disease, and end-stage renal disease were common in BSIs patients (50%, 43.8%, 10.9%, 9.4%, respectively). Only 26.6% of the patients had central venous access in the BSIs group. However, it did not differ significantly compared to non-BSIs patients (p-value = 0.31). Mechanical ventilation was used in 68.8% of patients with BSIs compared to 13.6% of non-BSIs patients (p-value = 0.001).

**Table 1:** Demographic, clinical and laboratory data of COVID-19 ICU patients (BSIs and non-BSIs group)

Parameters	All patients (n = 750)	Patients with BSIs (n = 64)	Non-BSIs (n = 686)	p value
Age (median)	62 years (IQR 51–72)	65 years (IQR 54–70)	60 (IQR 48–69)	0.31
(Mean ± SD)	60 ± 17.71 years	61.32 ± 13.74 years	59.13 ± 16.88 years	
Male (%)	562 (75)	42 (65.6)	487 (71)	0.37
q SOFA Score (median)		2		
Comorbidities and risk factors (%)				
Comorbidities^#^	317 (42.3)	44 (70)	273 (39.7)	0.01
Hypertension	183 (24.4)	32 (50)	151 (22)	
Diabetes mellitus	207 (27.6)	28 (43.8)	179 (26)	
Ischemic heart diseases	67 (8.9)	7 (10.9)	60 (8.7)	
Immunosuppressants use	64 (8.5)	9 (10.9)	55 (8)	
CKD/ESRD^$^	40 (5.3)	6 (9.4)	34 (5)	
Hypothyroidism	47 (6.3)	5 (7.8)	42 (6.1)	
Stroke	41(5.5)	4 (6.4)	37 (5.4)	
COPD	29 (3.9)	2 (3.2)	27 (3.9)	
Rheumatoid arthritis	23 (3.1)	2 (3.2)	21 (3.0)	
Chronic liver disease	29 (3.9)	1 (1.6)	28 (4.1)	
Invasive devices (insitu), (%)				
Foleys Catheter	485 (64.7)	46 (71.9)	439 (64)	0.20
Endotracheal intubation	137 (18.3)	44 (68.8)	93 (13.6)	0.001
Arterial line	282 (37.6)	21 (32.8)	261 (38)	0.24
Central venous line	251 (33.5)	17 (26.6)	234 (34.1)	0.31
Laboratory parameters, median (IQR)				
Hb (g/dl)	11.1 [8.8–12.9]	10 [8.25–12]	11. 3 [8.45–13.1]	0.51
Total leukocyte counts (10^3^/μl)	10.7 [7.73–15.91]	16.5 [11.2–22.8]	10.7 [7.73–15.91]	0.001
Platelet count (10^3^/μl)	247 [161–350]	198 [111–341]	273 [198–362]	0.10
CRP (mg/L)	100 [40.2–145.7]	128 [97–171]	96.1 [39.1–139.8]	0.06
Procalcitonin (ng/ml)	0.92 [0.145–6.99]	5 [1.75–15.5]	0.19 [0.09–1.12]	0.02
AST (IU/L)	48 [30–129]	44 [29–134]	49 [31–135]	0.73
ALT (IU/L)	37 [22–89]	42 [26–92]	36 [21–89]	0.60
Blood urea nitrogen (mg/dl)	48.5 [31–99.25]	100 [56–130]	35 [22–62]	0.002
Creatinine (mg/dl)	1.1 [0.84–1.8]	2 [1–3]	1.04 [0.84–1.5]	0.01
Bacterial microorganism identified in blood-stream				
*Acinetobacter baumannii*		21 (32.8)		
*Klebsiella pneumoniae*		14 (21.9)		
*Enterococcus* spp.		11 (17.2)		
*Escherichia coli*		7 (10.9)		
*Burkholderia cepaciae complex*		4 (6.3)		
*Pseudomonas aeruginosa*		4 (6.3)		
*Elizabethkingia* spp.		3 (4.7)		

*q SOFA* Quick Sequential Organ Failure Assessment; *CKD/ESRD* chronic kidney disease/End stage renal disease; *COPD* chronic obstructive pulmonary disease; *Hb* hemoglobin; *CRP* C reactive protein; *ALT* alanine aminotransferase; *AST* aspartate aminotransferase

^#^Some patients had more than one comorbid state

^$^Out of 40 patients, 14 patients needed hemodialysis support

Corticosteroids were used in all patients, including non-BSIs patients (as a part of the standard treatment regimen of severe COVID-19). Seven patients received tocilizumab for COVID-19 pneumonia and developed BSIs. Tocilizumab use was not associated with incidence of BSIs (12.3% vs 8.2% in tocilizumab vs non-tocilizumab group, p-value = 0.29). Two patients were renal transplant recipients and receiving tacrolimus during hospitalization. Piperacillin-tazobactam (n = 42) was the most common empirical antibiotic used, followed by Levofloxacin (n = 7) and Ceftriaxone (n = 5). Subsequently, antibiotics were changed based on the blood culture sensitivity report and clinical improvement.

**Fig. 1:**
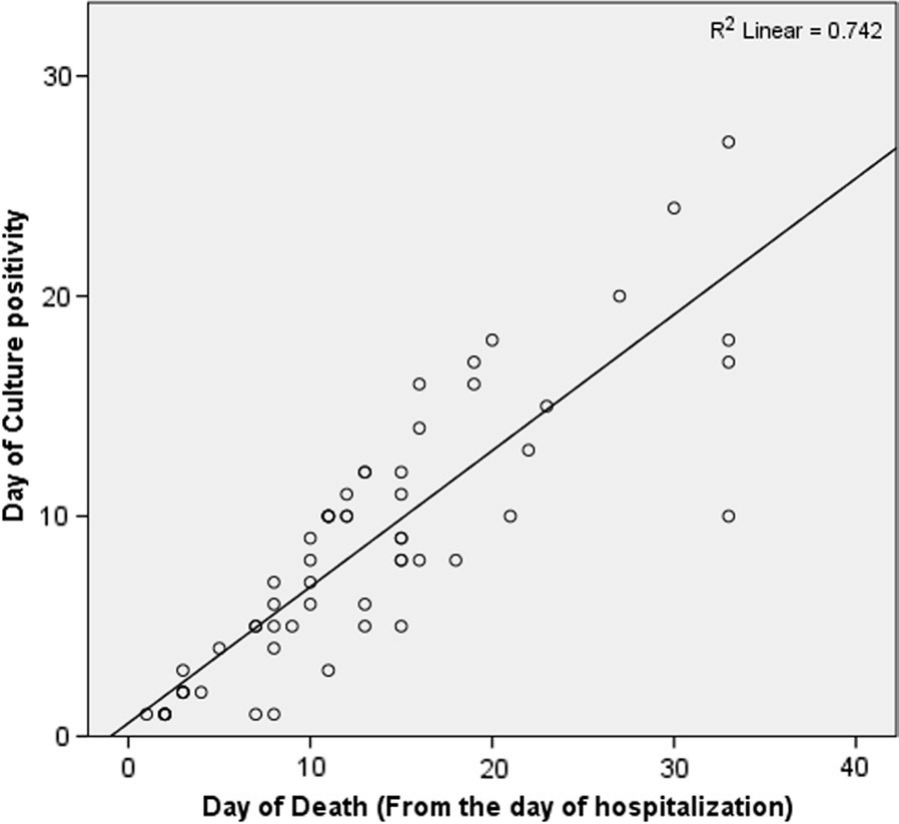
Linear regression graph showing positive correlation between day of culture positivity and day of mortality

Biochemical parameters showed significant elevated CRP and procalcitonin levels in BSIs patients on admission (median CRP 128 mg/L and procalcitonin 5 ng/ml, Table 1). The median time from onset of symptoms to ICU admission was 5 days (IQR 3–7), and the median time from ICU admission to the first BSI episode was 8 days (IQR 4–11.8). The overall mortality rate was 100% (64) in the BSIs group and 33.8% (232) in the non-BSIs group, which was statistically significant (p-value < 0.0001). The patients who died in the hospital spent a median of 11.5 days (IQR 7.25–16 days). Out of 64 patients with BSIs, 43 developed septic shock, and three patients had features of septic encephalopathy. We analyzed the role of BSIs and sepsis in the clinical outcome of COVID-19 patients admitted to ICU. Linear regression analysis showed a positive correlation (coefficient of determination, R2 = 74.2%) between time to positivity of blood cultures and time to death (from the day of hospitalization) after adjusting confounding factors like age and sex (Fig. 1). Univariate analysis showed a significant association of procalcitonin level, leukocytosis and presence of comorbidities with BSIs (Table 2). Further multivariate analysis revealed comorbidities, mechanical ventilation, and raised leukocyte counts as an independent predictor of BSIs (Table 2).

**Table 2:** Different variables and their associations with the incidence of BSIs in Covid-19 ICU patients in the univariate and multivariate analysis

Characteristics	Univariate analysis		Multivariate analysis	
	OR (95% CI)	p value	OR (95% CI)	p value
Age (> 60 years)	1.0 (0.98–1.2)	0.36		
Gender (Male)	0.78 (0.36–1.7)	0.53		
CRP (> 6 mg/L)	1.3 (0.97–1.8)	0.31		
Procalcitonin (> 0.05 ng/ml)	1.8 (0.89–2.7)	0.04	1.5 (0.78–2.2)	0.12
Comorbidities^a^	3.2 (1.6–6.3)	0.003	2.9 (1.6–5.8)	0.042
Fever	1.7 (0.86–3.3)	0.129		
TLC (> 14 × 10^3^/μl)	4.2 (2.8–8.9)	0.006	3.7 (2.6–7.10)	0.01
Immunomodulatory drugs (Tocilizumab)	1.4 (0.54–2.1)	0.29		
Mechanical Ventilation	9.0 (4.7–17.4)	< 0.001	4.1 (2.9–10.4)	0.001

*CRP* C reactive protein, *OR* odds ratio, *CI* confidence interval

^a^Patient with comorbid conditions which could be single or combination of more than one disease, refer to Table 1

### Characteristics of microorganisms and antimicrobial resistance profile associated with BSIs

**Fig. 2:**
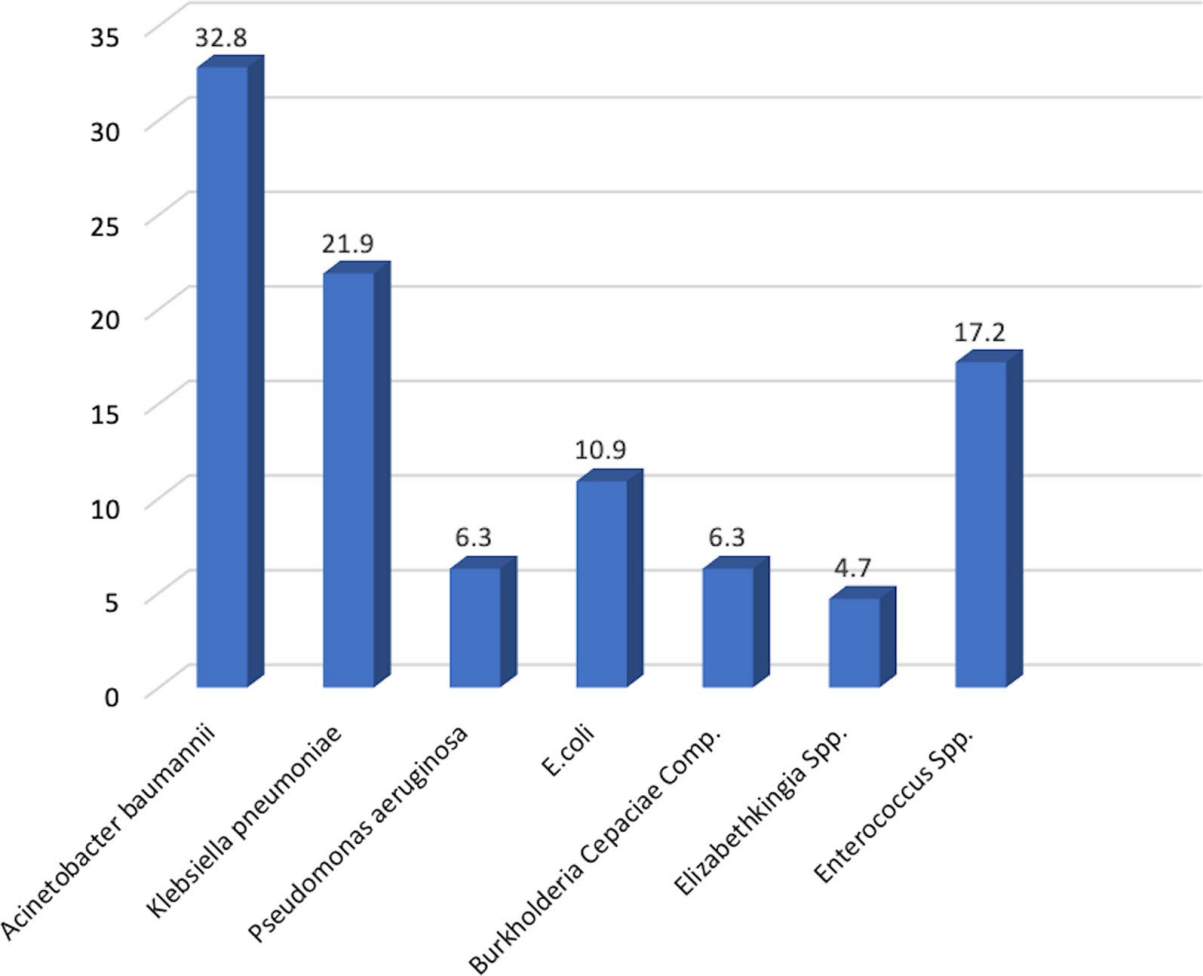
Frequency and distribution of pathogenic organism isolated from COVID-19 ICU patients (%)

All of the BSIs were monomicrobial in this study (Table 1). The majority of the isolates were Gram-negative microorganisms (53/64, 82.8%). Among Gram-positive microorganisms, all isolates were from the Enterococcus group (11/64, 17.2%). The most common Gram-negative bacteria (GNB) were *Acinetobacter baumannii* (32.8%, 21/64), and *Klebsiella pneumoniae* (21.9%, 14/64). The remaining Gram-negative microorganisms (17.2%) were *Burkholderia cepaciae complex*, *Pseudomonas aeruginosa*, and *Elizabethkingia* spp. (Fig. 2). None of the patients had polymicrobial BSIs (isolation of > 1 microorganism from the bloodstream) in this study. Out of sixty-four patients, 57.8% patients were infected with MDRO. The majority of MDRO belonged to *K. pneumoniae* and *Enterococcus* spp. The incidence of Carbapenems resistance Gram-negative bacteria (CR-GNB) was 47.2% (25/53).

Among isolated pathogens, the highest resistance for *Acinetobacter baumannii* was observed with ceftriaxone (76.2%), and piperacillin-tazobactam (76.2%, Table 3). *Klebsiella pneumoniae* isolates showed the highest resistance rate against aztreonam (85.7%). All *E. coli* isolates were resistant to ceftriaxone (100%). Among Gram-positive organisms, *Enterococcus* spp. showed maximum resistance for erythromycin, and ampicillin (90.9%, and 81.8%, respectively), (Table 4).

**Table 3:** Antibiotic resistance pattern of predominant Gram-negative blood stream isolates in Covid-19 ICU patients (%)

Antibiotic	*Acinetobacter baumannii* (n = 21)	*Klebsiella pneumoniae* (n = 14)	*Psuedomonas aeruginosa* (n = 4)	*Escherichia coli* (n = 7)	*Burkholderia cepacia complex* (n = 4)	*Elizabethkingia* spp. (n = 3)
Ceftriaxone	76.2	71.4	NA	100	NA	NA
Cefepime	52.4	78.6	0	85.7	NA	66.6
Ceftazidime	NA	NA	NA	NA	50	NA
Piperacillin-Tazobactam	76.2	78.6	0	71.4	NA	33.3
Meropenem	33.3	21.4	0	14.3	75	100
Colistin	9.5	7.1	0	14.3	50	33.3
Ciprofloxacin/Levofloxacin	66.7	78.6	NA	85.7	50	NA
Cefoperazone/sulbactam	NA	71.4	0	57.1	NA	NA
Amikacin	52.4	57.1	0	57.1	NA	66.6
Tigecycline	0	0	NA	0	NA	NA
Aztreonam	NA	85.7	50	71.4	NA	66.6
Imipenem	9.5	NA	0	28.6	NA	NA
Ertapenem	23.8	71.4	NA	NA	NA	NA

**Table 4:** Antibiotic resistance pattern of predominant Gram-positive blood stream isolates in Covid-19 ICU patients (%)

Antibiotic	Enterococcus spp. (N = 11)
Ampicillin	81.8
Tigecycline	0
Ciprofloxacin	81.8
Tetracycline	54.5
Erythromycin	90.9
Linezolid	0
Teicoplanin	18.1
Vancomycin	18.1

## Discussion

The emerging data regarding the secondary bacterial infections in COVID-19 are scarce and conflicting, even more so with BSIs. In this study, we found the overall prevalence of BSIs in ICU patients to be 8.5%. A recent study from India found the prevalence of secondary infections to be 3.6% [16]. However, in their report, both hospital-acquired and community-acquired cases were taken. In addition, apart from BSIs, respiratory specimens were also analyzed, which could be contaminants or colonizers. Another report by Khurana et al. showed a 13% prevalence of secondary infections [17]. Contrary to previous studies, Lai et al. described the prevalence of BSIs up to 50% among non-survivors severe COVID-19 pneumonia patients [18]. Further large prospective studies are needed to determine the precise prevalence of BSIs in severe COVID-19 and its implications on the outcome of patients.

Interestingly, we found a 100% mortality rate in severe COVID-19 pneumonia patients with BSIs. Previous studies have reported 21–68% mortality in this group of patients [4, 5, 16, 19]. The disparity in mortality rate could be due to various factors, first of all the severity of the COVID-19 illness and the need for ICU admission. In previous studies, other sources of superinfection were analyzed e.g.: respiratory, urinary tract, and local tissue samples with a higher possibility of contaminations. Furthermore, the follow-up period was in some studies was brief or incomplete. Comorbidity also played an important role in mortality, with nearly half of the patients in this study having diabetes and hypertension. Few studies also showed high COVID-19 associated mortality in the male gender [20, 21]. However, our report did not find a similar result. Finally, we found sepsis as a determining factor in clinical outcomes, with a temporal relationship between sepsis/septic shock and mortality.

In this report, we found significantly elevated CRP and procalcitonin levels in patients who developed BSIs. However, the utility of inflammatory markers for predicting BSIs and empirical initiation of antibiotics in these patients remains debatable. Few studies reported the poor correlation of serum procalcitonin and CRP levels with bacterial coinfections, especially in the setting of immunomodulatory/immunosuppressive therapy [5, 19, 22]. Kreitmann et al. reported that median CRP and procalcitonin did not differ significantly in patients with or without bacterial coinfections (median procalcitonin, 0.4 vs 0.72 ng/ml, CRP, 182 vs 159 mg/L) [23]. Although procalcitonin levels were significantly raised in the BSIs group, we did not find an independent association between the incidence of BSIs and procalcitonin and other inflammatory markers (CRP). The inflammatory markers should be interpreted cautiously in COVID-19 before the initiation of empirical antibiotics.

Gram-negative microorganisms were predominant BSIs in this report. Similar observations were described by a multicentric study from India with a predominance of Gram-negative pathogens (78%) [16]. Conversely, Elabaddi et al. and a few other studies reported the increased prevalence of Gram-positive microorganisms, particularly *Staphylococcus aureus* (prevalence varies from 44 to 79.6%) in COVID-19 ICU patients [5, 19]. This heterogenicity in prevalence and distribution of microorganisms may attribute to different patient settings, the number of patients on mechanical ventilation, duration of hospital stays and follow-up and isolation of the pathogen from other specimens (like respiratory, urine and pus samples) in addition to BSIs. In this study, we observed the relatively high proportion of *Enterococcus* isolates as BSI, and the majority were multidrug-resistant (81.8% MDRO). Bonazetti et al. proposed a theory that SARS-CoV-2 mediated disruption of the gut barrier and bacterial translocation could trigger increased BSIs, especially *Enterococci* spp. [4]. Genotypic analysis was not available at our Centre, which would have provided some insight into this occurrence. Conflicting evidences are emerging regarding the prevalence of MDRO in the COVID-19 pandemic. Few reports showed a decreased prevalence of MDRO due to effective implementation of infection control practices; in contrast, there are studies that showed a high transmission of MDRO due to prolonged ICU stay and use of multiple antibiotics in COVID-19 [24–26].

In this study, we also observed the high prevalence of *A. baumannii* (32.8%). However, previous reports described *A. baumanni* in < 1% of the cases [4, 27, 28]. The other worrying aspect was the high proportion of CR-GNB in these isolates (nearly 50%). This increase in prevalence and resistance could be explained by prolonged ICU stay, mechanical ventilation, central venous catheter use, and inadvertent use of carbapenem with antimicrobial selection pressure, which are the usual triggers [29, 30]. Moreover, inadequate compliance with infection control procedures (like hand hygiene, disinfection of hospital equipment and environment) is another vital factor contributing to multidrug resistance organism infections.

The appropriateness of the use of empirical antimicrobials in severe COVID-19 patients is a matter of intense debate. According to a recent systematic review, the prevalence of empirical antibiotic use in COVID-19 patients was 74% [31]. Which was lower than our study (> 90% in our report); however, the pool of the patients in that review also included the mild to moderate COVID-19 patients. Despite the increasing use of empirical antibiotics, there is a lack of data favouring their use. Chedid et al. did not found a significant difference in antibiotic use among survivors and non-survivors COVID-19 patients [31]. Furthermore, exposure to antimicrobials increases the risk of drug resistance. According to a recent study, the use of combination antimicrobials was found to be associated with secondary infections [32]. Future prospective studies are needed to validate the aforementioned points before reaching the consensus regarding empirical antibiotic use in COVID-19.

This study has a number of limitations; first, this was a retrospective analysis which probably led to selection bias and inclusion of severe patients, which may have impacted the clinical outcome. Secondly, data were from a single centre, which may preclude its generalized applicability. All patients received corticosteroids and other immunosuppressants resulting in a high prevalence of secondary bacterial infections. In addition, the information about prognostic markers (e.g. SOFA, APACHE score) and central line days (central line insertion/1000 catheter days) was also lacking. Lastly, the lack of prospective design precluded the genotypic analysis of MDRO (*A. baumannii*), which is vital in view of patient cross-contamination in ICU settings.

## Conclusions

BSIs in severe Covid-19 are associated with poor outcome. We report a high prevalence of *Acinetobacter baumannii* BSIs in the ICU setting. The presence of comorbidities and leukocytosis should alert the clinicians for possible BSIs. At the same time, clinicians should be prudent while interpreting the results of CRP and procalcitonin. We encourage implementing Antimicrobial stewardship and infection control practices, which could reduce the secondary bacterial infections in COVID-19 illness. Further prospective studies are warranted to determine the precise burden of secondary bacterial infections and their impact on mortality and morbidity in COVID-19.

## Data Availability

The datasets used and/or analysed during the current study available from the corresponding author on reasonable request.

## Abbreviations

BSIs: Bloodstream infections
Covid-19: Corona virus disease
RT-PCR: Reverse-transcription polymerase chain reaction
ICU: Intensive care unit
MDRO: Multidrug-resistance microorganisms
CLABSI: Central line-associated blood stream infections
CRP: C-reactive protein
LDH: Lactate dehydrogenase
q-SOFA: Quick sequential organ failure assessment
ESRD: End stage renal disease
GNB: Gram-negative bacteria
TGF-β: Transforming growth factor-β
CR-GNB: Carbapenems resistance Gram-negative bacteria
